# Online Problem-Based Learning in Clinical Dental Education: Students’ Self-Perception and Motivation

**DOI:** 10.3390/healthcare9040420

**Published:** 2021-04-05

**Authors:** Mariana Morgado, José João Mendes, Luís Proença

**Affiliations:** 1 Clinical Research Unit (CRU), Centro de Investigação Interdisciplinar Egas Moniz (CiiEM), Egas Moniz—Cooperativa de Ensino Superior CRL, Campus Universitário, Quinta da Granja, 2829-511 Caparica, Portugal; mmorgado@egasmoniz.edu.pt (M.M.); jmendes@egasmoniz.edu.pt (J.J.M.); 2 Evidence-Based Hub, CiiEM, Egas Moniz—Cooperativa de Ensino Superior CRL, Campus Universitário, Quinta da Granja, 2829-511 Caparica, Portugal; 3 Quantitative Methods for Health Research (MQIS), CiiEM, Egas Moniz—Cooperativa de Ensino Superior, CRL, 2829-511 Almada, Portugal

**Keywords:** problem-based learning, dental education, e-learning, innovation in teaching, clinical teaching

## Abstract

The physical closure of higher education institutions due to coronavirus disease 2019 (COVID-19) shed a brighter light on the need to analyze, explore, and implement strategies that allow the development of clinical skills in a distance learning situation. This cross-sectional study aims to assess dental students’ self-perception, motivation, organization, acquired clinical skills, and knowledge using the online problem-based learning method, through the application of a 41-item questionnaire to 118 senior students. Answers were subjected to descriptive and inferential statistics analysis. Further, a principal component analysis was performed, in order to examine the factor structure of the questionnaire. Results show that online problem-based learning can be considered a relevant learning tool when utilized within the specific context of clinical dental education, displaying benefits over the traditional learning strategy. Overall, dental students prefer a hybrid system over the conventional one, in a distance learning context, and assume self-responsibility for their own learning, while knowledge thoroughness is perceived as inferior. This online active learning method is successful in improving information and clinical ability (visual/spatial and auditory) advancement in the scope of dental education, with similar results to presential settings. Further studies are required to assess clinical skill development through active learning methods, in a distance learning context.

## 1. Introduction

Education is rooted in a social context, in particular its innovation. In the post-war era of the 1960s and 1970s, in line with the new values of cognitive psychology, passive learning and the reward concepts of behaviorism have been replaced by active, student-centered learning, with growing prominence given to individuality, equality, and personal development rather than authority and deductive learning. Problem-based learning (PBL) was part of this context of change [1,2].

Medical education has long been a stronghold of conventional methods. Nonetheless, in the aforementioned period, there were many changes in this specific field that led to the inevitable reform and innovation in the teaching of prospective medical practitioners, which included social critique, the rise and fall of clinical medicine, as well as the volume and changeability of medical knowledge [3,4].

Problem-based learning originated in 1966, at McMaster University, Canada [5], and groundbreaking work was later carried out at Newcastle University (Australia), Michigan State University (United States), and Maastricht University (The Netherlands) [6]. The first PBL-based medical curriculum was introduced in 1969 at McMaster University, while only in 1990 did the first PBL-based dental curriculum arise at the Faculty of Odontology in Malmö, Sweden [7].

While there are parallels between medical and dental education, there is a need to emphasize that the qualifications students need to learn are not exactly the same. Clinical skills in medical education, for example, are generally defined as clinical thinking and problem-solving, both of which are connected to doing and accurate physical assessment and performing a correct diagnosis [8]. While these clinical skills are also important for dental students to acquire, there is a need to further develop visual/spatial, auditory, and kinesthetic skills [9], which sums up to the point that dental education is quite different from medical education, mainly in the pre-clinical and clinical years [10].

Advances in technology have led to changes, not only inevitable but exponential [11], at all levels of education, but namely in higher education [12]. Emerging technologies, by allowing ubiquitous connections between individuals, content and digital/intelligent objects, in addition to enhancing learning [13], have created new teaching–learning dynamics, where students have different needs depending on the specific area of training, for example, the health area [14,15].

In the context of medical and medical–dental education, this paradigm shift has been explored through the implementation of active learning methods [16], such as self-guided study [17]; problem-based learning (PBL) [18]; mixed learning strategies, such as blended learning (BL) [19]; using asynchronous digital tools (e-Learning); and “inverted” or flipped classrooms [20], using didactic material previously available online, as well as simulation instruments [21] and gamification [22].

With the advent of the coronavirus disease 2019 (COVID-19) pandemic that we currently live in, and the physical closure of higher education institutions (HEIs), it is necessary to analyze, explore, and implement strategies that allow the development of clinical skills, even in face of a distance learning situation [23,24,25].

This study was aimed to assess dental students’ self-perception on learning, motivation, organization, tool acquisition, clinical skills, and knowledge using the PBL method, through online/digital channels in a distance learning context, as well as the identification of limitations and difficulties in this context.

## 2. Materials and Methods

### 2.1. Course Description

**Figure 1:**
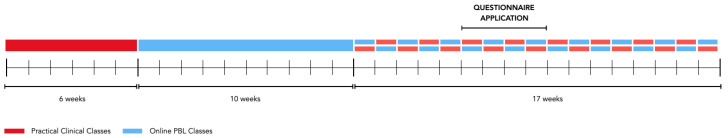
Dentistry Integrated Master Course final year schedule (33 weeks). Every space represents a week. Red boxes represent practical (presential) clinical classes. Blue boxes represent online problem-based learning (PBL) classes.

The participants of this cross-sectional study, students of the Dentistry Integrated Master Course at Instituto Universitário Egas Moniz (IUEM), a higher education institution located in the southern Lisbon Metropolitan Area (Portugal), were enrolled during the fifth and final, year of their course. The study took place during the 2019/2020 term, from March to September 2020. In Portugal, by March 2020, a national lockdown was imposed due to the COVID-19 pandemic. As a result, from that date, higher education schools had to close and started teaching exclusively online, through digital platforms. This exclusively online teaching lasted for 10 weeks. Thus, the final year of the Dentistry Integrated Master Course at IUEM, during the 2019/2020 term, spanned over 33 weeks and was divided in three periods, according to the type of teaching that was implemented: six fully clinical (presential) weeks, 10 fully online synchronous weeks, and 17 hybrid (presential/online) weeks, as depicted in Figure 1.

During clinical practice weeks, students underwent six hours of live-patient encounters (LPE) *per* day at the University Dental Clinic. On online PBL weeks, students had six synchronous hours of real clinical case presentation and discussion *per* day. On mixed weeks, half of the students had clinical practice, while the other half had online lectures. Every week, the student groups switched, as indicated in the schedule (Figure 1).

### 2.2. Study Design

The study was conducted through the application of an online questionnaire, via Google Forms. All of the 211 senior dental students of the Dentistry Integrated Master Course at IUEM were enrolled to participate, from which 118 (55.9%) answered the questionnaire. The exclusion criteria for participating in the study were as follows: refusal to provide informed consent and being enrolled in other university study programs. None of the students that answered the questionnaire were excluded. 

**Figure 2:**
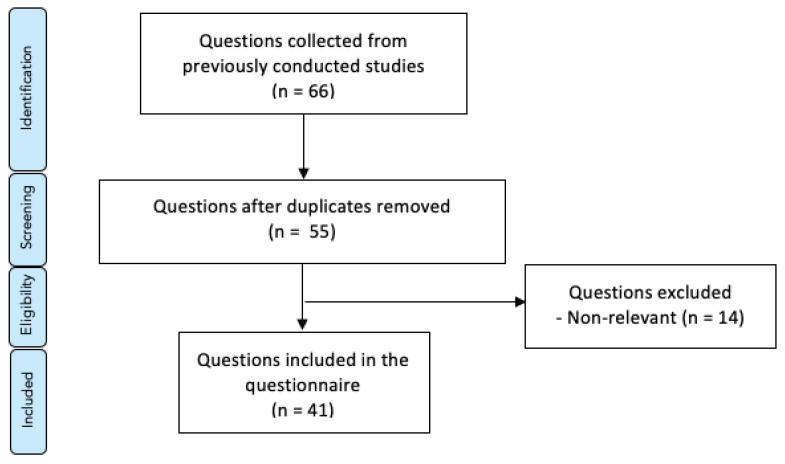
Preferred Reporting Items for Systematic Reviews and Meta-Analyses (PRISMA) flow chart. Presentation of the procedure of question selection with number of questions at each stage.

A 41-item online questionnaire was constructed after a detailed literature review by using web-based search engines like PubMed and Google Scholar. The keywords “problem-based learning”, “conventional lectures”, “medical education”, “dental education”, “clinical education”, and “student’s perception” were used to search the literature. The final group of 41 close-ended questions were assembled from a 66-item database, collected from previously conducted surveys [26,27,28,29,30,31]. Exclusion criteria for the questions included lack of relevance to the scope of the study and duplicates, as shown in Figure 2. The content validity and clarity of the questionnaire was ensured by a review done by experts in medical education. Answers were coded following a five-point Likert rating scale (1 = “strongly disagree”, 2 = “disagree”, 3 = “neither agree nor agree”, 4 = “agree”, 5 = “strongly agree”).

The description of the 41 items/questions included in the online questionnaire are presented in Table 1.

### 2.3. Ethical Considerations

The questionnaire was sent directly, via e-mail, to each one of the participants, from a third party not involved in the present study. The anonymous, voluntary, self-completion questionnaire was preceded by informed consent, which had to be provided in order to participate. The present work is part of an ongoing research project regarding the implementation and evaluation of learning methodologies for clinical dental teaching, approved by the Scientific Council of IUEM.

### 2.4. Statistical Analysis

Data analysis was performed using IBM SPSS Statistics software version 26.0 for Windows (IBM Corp., Armonk, NY, United States). Descriptive and inferential statistics methodologies were applied. Furthermore, a principal component analysis (PCA) was performed to examine the factor structure of the questionnaire. 

All of the items were distributed, according to the rotated component matrix, via Varimax, with Kaiser normalization methods. The obtained Kaiser–Meyer–Olkin (KMO) validity test value was 0.902, falling into the range between 0.8 and 1.0, that deems a sample adequate for factor analysis. The extraction and retention of factors were based on visual examination of the scree plot, and eigenvalues >1.0 were retained. Factor loadings approximately equal to or higher than 0.50 dictated the assignment to a certain component. For questions with similar scores, a decision was made upon the contextualization of the question within each component, thus making its assignment to each one of two components possible.

## 3. Results

The mean age of the respondents was 28.7 (±7.6) years, and the majority (65.3%) were females (Table 2). Although 11% were not native Portuguese speakers, those participants had full understanding of the language, since the great majority (96%) self-reported an advanced (C1: 20%) or proficient (C2: 76%) level of Portuguese language reading skills. 

The results of the answers to the 41-item questionnaire, based on a five-point Likert scale, are presented in Table 3. In general, a relatively high score was obtained for most of the questions (median, equal to, or higher than 4.0). The highest values were recorded for questions Q08 (student knowledge responsibility), with a median of 5.0, followed by Q22 (comparison of the PBL hybrid system with the conventional method), with a median of 4.5. The lowest score was obtained for questions Q11 (kinesthetic learning in a clinical context), Q23 (comparison of knowledge thoroughness gain), Q26 (time consumption comparison), and Q27 (effectiveness comparison), with a median of 3.0.

When comparing the answers as a function of gender, no statistically significant differences were found (*p* > 0.05, Mann–Whitney test).

The PCA results are presented in Table 4. Eight components were identified, according to the established criteria. As presented, in Component 1, Q13 (learning motivation, in a clinical context), Q14 (clinical interest in the lectured lessons), Q24 (comparison of relevance and interest), Q19 (clinical problem-solving ability), Q25 (comparison of understanding of objectives), Q27 (effectiveness comparison), Q23 (comparison of knowledge thoroughness gain), Q30 (establishment of a concrete action plan for the achievement of learning goals), and Q29 (identification of weakness areas) were categorized. In Component 2, Q01 (PBL interest), Q03 (use of learning resources for clinical learning), Q06 (clinical exam preparation), Q04 (achievement of curriculum outcomes), Q05 (understanding of basic concepts), Q02 (interactive clinical learning environment), Q07 (subject understanding), Q41 (extension of related knowledge), and Q40 (knowledge retention by practice, feedback, and evaluation) were categorized. In Component 3, Q16 (ability for public speaking), Q20 (development of linguistic skills and self-confidence in a clinical context), Q15 (ability to find information using the internet/library), Q17 (time-management skills), Q18 (decision-making skills), Q21 (clinical reasoning ability in a clinical context), and Q35 (development of scientific reading and writing skills) were categorized. In Component 4, Q33 (horizontal integration effectiveness), Q34 (vertical integration effectiveness), Q31 (practical and clinical application of ideas), Q32 (development of clinical thinking, logical thinking, and abstract concepts), Q39 (knowledge organization), Q38 (prior knowledge activation around a problem), and Q22 (comparison between the PBL hybrid system and the conventional method). In Component 5, Q09 (visual/spatial learning in a clinical context), Q12 (conversion to active lifelong learner in a clinical context), Q11 (kinesthetic learning in a clinical context), and Q10 (auditory learning in a clinical context) were categorized. In Component 6, Q36 (development of interpersonal skills) and Q37 (development of intrapersonal skills) were categorized. In Component 7, Q08 (student knowledge responsibility) and Q28 (active processing of information) were categorized. In Component 8, a single question was categorized: Q26 (time consumption).

In ascending order, the percent of Component 1 for total variance was approximately 49.96%, Component 2 was approximately 5.61%, Component 4 was 3.98%, Component 5 was approximately 3.90%, Component 6 was 3.21%, Component 7 was approximately 2.69%, Component 8 was approximately 2.47%, and Component 3 was approximately 1.99%. The cumulative variance of total factors was approximately 73.64%.

## 4. Discussion

Amid the lockdown imposed due to the COVID-19 pandemic, the physical closure of higher education institutions (HEIs) has shed a brighter light on the need of pedagogical innovation. Given the specificity of medical and dental education, this crisis is necessary to analyze, explore, and implement strategies that allow the development of clinical skills, even in face of a distance learning situation.

The present study was aimed at assessing dental students’ self-perception on learning, motivation, organization, tool acquisition, clinical skills, and knowledge using an active learning method, such as PBL, through online/digital channels in a distance learning context, as well as the identification of the limitations and difficulties in this context.

Given the primacy of the present investigation, all of the results were compared to presential PBL method studies.

When comparing the answers as a function of the gender, no statistically significant differences were found (*p* > 0.05, Mann–Whitney test), contrary to previous evidence [27].

Concerning the items categorized to Component 1, 63.6% of the students agreed that the PBL method increased learning motivation in a clinical context (Q13), which supports previously conducted studies that reported 65.2%, but is inferior to those reported before of 95.6% [28] and 88.0% [30]. As for Q14, 71.2% agreed that the PBL method helps to create clinical interest, which supports previous evidence, but nonetheless is inferior to 95.6% reported [28]. Of the responding students, 71.2% agreed that the PBL method enhances clinical problem-solving abilities (Q19), which is in line with previously conducted study values of 66.7% [27] and 73.2% [31]. All of the remaining items from this component did not support previous evidence. As to comparing the knowledge thoroughness gained by the PBL method versus the conventional method (Q23), only 36.4% of the present study students agreed, which is inferior to the percentage of 53.3% previously reported [27]. The same tendency is verified in Q24 (comparison of relevance and interest), with 67.8% versus 83.4% previously [27]. When comparing to the subjects’ understanding of objectives to the conventional method (Q25), 58.5% of the students of the present study agreed that the PBL method is better, facing 43.4% reported before [27]. As to the PBL method’s effectiveness without a conventional lecture (Q27), 46.6% agreed, while only 20.0% was reported in a previous study [27]. Also, in Q29, while assessing if the PBL method helped identify areas of weakness for further improvement, our results found that 72.0% agreed, while only 50.0% was reported in a previous study [27].

The majority of the items categorized in Component 2 support previous evidence, namely PBL interest (Q01: 89.0% vs. 70.0% [27]), interactive clinical learning environment (Q02: 81.4% vs. 79.5% [31]), use of learning resources for clinical learning (Q03: 76.3% vs. 82.1% [31]), achievement of curriculum outcomes (Q04: 63.6% vs. 65.2% [31]), and extension of related knowledge (Q41: 83.1% vs. 83.0% [31]). As for Q40, 81.4% of the students agreed that the PBL method enhances knowledge retention by practice, feedback, and evaluation, which is consonant to what has been reported—85.7% [31]—but contrary to other results of 60.0% [30]. In what concerns Q07 (subject understanding), the results of the present study find that 76.3% agreed that the PBL method allows a better understanding about the subject, which is inferior to previous results of 97.2% [28]. This question was also mentioned in other previous studies, and even though strict data comparison is not possible. Due to the ordinary nature of the Likert scale, results (3.87 ± 0.79) are inferior to those presented of 4.42 ± 1.13 [26] and higher than 3.23 ± 0.57 [29]. Regarding Q05 (understanding basic concepts), our results (3.97 ± 0.78) are inferior to those previously reported of 4.24 ± 0.88 [26].

Most of the items categorized in Component 3 did not support previous evidence, with results inferior to those reported, namely ability to find information using the internet/library (Q15: 61.0% vs. 90.0% [27]), ability for public speaking (Q16: 52.5% vs. 93.3% [27] and 77.6% [26]), time-management skills (Q17: 55.9% vs. 90.1% [27] and 90.6% [26]), clinical reasonability in a clinical context (Q21: 76.3% vs. 90.6% [26]), and development of scientific reading and writing skills (Q35: 68.6% vs. 80.3% [31]). The exceptions for this tendency can be observed in Q18 (decision-making skills), where 66.1% of the students agreed that the PBL method helped in developing linguistic skills and self-confidence, in accordance with 60.0% [27] and 73.2% [31] previously, as well as in Q20 (development of linguistic skills and self-confidence in a clinical context), where the results obtained by the present study (58.5%) are superior to those reported earlier (48.5% [31]).

The majority of the items categorized in Component 4 support previous evidence, namely the practical and clinical application of ideas (Q31: 64.4% vs. 73.2% [31]); development of clinical thinking, logical thinking, and abstract concepts (Q32: 81.4% vs. 83.0% [31]); and prior knowledge activation around a problem (Q38: 76.3% vs. 73.3% [27] and 83.0% [31]). Regarding horizontal integration effectiveness (Q33: 86.4%), vertical integration effectiveness (Q34: 78.0%), and knowledge organization (Q39: 84.7%), our results were higher than those reported of 75.0% [31], 60.7% [31], and 73.3% [27], respectively. In regards to Q22, 81.4% of the students of the present study agree that a PBL hybrid system is better than an exclusively conventional method, which is a significantly higher percentage than the reported value of 46.4% [27].

Regarding the items categorized in component 5, 66.9% of the students agreed that the PBL method can facilitate auditory learning in a clinical context (Q10), and only 39.0% agreed on the facilitation of kinesthetic learning, which supports previous evidence of 79.5% [31] and 44.7% [31], respectively. The results obtained in Q9, visual/spatial learning in a clinical context (71.2%), support those reported (76.5%) from a previous study [26], while being superior to other results of 55.4% [31]. In relation to Q12, 70.3% of the students of the present study agree that the PBL helps the conversion from a passive to active lifelong learner in a clinical context, which is a significantly higher percentage than the 50.0% reported previously [27].

All of the items categorized in Component 6 did not support previous evidence, showing inferior results in Q36, development of interpersonal skills (53.4%), and Q37, development of intrapersonal skills (61.0%), to those reported in previous studies of 88.4% [31] and 87.5% [31], respectively.

Meanwhile, all of the items categorized in Component 7 support previous evidence, showing significantly higher results in Q8, student knowledge responsibility (90.7%), and Q28, active processing of information (83.1%), than those reported in previous studies of 70.0% [27] and 63.0% [27].

The only item categorized in Component 8 was not comparable, since the previous conducted study [32] compared all items as one, instead of an isolated analysis.

These findings deserve further reflection, hence the fact that even if there are other goals that can be accomplished with PBL, clinical reasoning (Q21), self-directed learning (Q08), and most of the factors referred to in Component 4 represent essential medical skills, while students’ motivation (Q13) expands their internal learning drive, thus encouraging extraction and comprehension of data from learning platforms (Q03). All of these variables have been tested in the present study, and have shown positive results in online PBL settings. Regarding the four domains of VARK (visual, auditory, reading/writing, and kinesthetic), which represent essential skills for the dental field, our results demonstrate that PBL, even in a distance learning context, can facilitate visual/spatial, auditory, and reading/writing domains, while not easing the development of the kinesthetic domain due to the lack of tools that allow at-home, hands-on practice. Concerning the comparison to conventional methods in this particular setting, students self-perceived benefiting in regards to knowledge thoroughness (Q23) and subjects’ understanding of objectives (Q25), while considering online PBL to be more relevant and interesting (Q24). This summed up to a majority belief that the hybrid method is better than the conventional one (Q22), with significantly superior results in this setting (81.4%), than in a previous one (46.4%) [27].

Several limitations must be taken into account when interpreting the findings. The most important is that the present study has a low response rate and is limited as to the sample size, which may not fully allow generalization for the whole population of dental students.

## 5. Conclusions

This study’s results demonstrate that online PBL can be considered a relevant learning tool to be utilized within the specific context of clinical dental education, displaying benefits over traditional learning strategies.

Overall, dental students prefer a PBL hybrid system over the conventional one, in a distance learning context, and assume self-responsibility for their own learning. On the other hand, knowledge thoroughness is perceived as inferior to that gained through the conventional method.

The online PBL method is not a reliable tool to facilitate kinesthetic learning in a dental clinical setting, as compared to other methods.

Nonetheless, the online PBL strategy can be viewed as a successful instrument to improve information and clinical ability (visual/spatial and auditory) advancement in the scope of dental education, with similar results to those obtained in a presential setting.

Further studies are needed to assess clinical skills and knowledge development through active learning methods, such as PBL, in a distance learning context, as well as to design and implement effective complementary kinesthetic distance learning tools, namely through haptic technology, and also with a greater focus on the development of communication and personal skills, such as video-based online approaches, that have been showing promising results.

## Figures and Tables

**Table 1:** Items included in the online questionnaire.

ID	Item
Q01	PBL is interesting.
Q02	PBL provides an interactive clinical learning environment.
Q03	PBL facilitates the use of learning resources for clinical learning.
Q04	PBL helps the achievement of curriculum outcomes.
Q05	PBL helps understand basic concepts.
Q06	PBL helps clinical exam preparation.
Q07	PBL helps to have a better understanding about the subject.
Q08	With PBL, students assume responsibility for their own learning.
Q09	PBL is a reliable tool that can facilitate visual/spatial learning in a clinical context.
Q10	PBL is a reliable tool that can facilitate auditory learning in a clinical context.
Q11	PBL is a reliable tool that can facilitate kinesthetic learning in a clinical context.
Q12	PBL helps convert from a passive to active lifelong learner in a clinical context.
Q13	PBL increases the learning motivation in a clinical context.
Q14	PBL helps to create clinical interest in the lectured lessons.
Q15	PBL enhances the ability to find information using the internet/library.
Q16	PBL enhances ability for public speaking in the clinical context.
Q17	PBL increases the ability to manage time effectively in the clinical context.
Q18	PBL improves decision-making skills in the clinical context.
Q19	PBL enhances clinical problem-solving ability in the clinical context.
Q20	PBL helps develop linguistic skills and self-confidence in the clinical context.
Q21	PBL enhances clinical reasoning ability in the clinical context.
Q22	A PBL hybrid system, composed by joining PBL and the conventional learning methods, is better than an exclusively conventional learning method.
Q23	When compared to the exclusively conventional learning method, the knowledge achieved with PBL is more thorough.
Q24	When compared to the exclusively conventional learning method, the focus of PBL on real medical/dental cases, makes it more relevant and interesting.
Q25	When compared to the exclusively conventional learning method, the subject objectives are better understood with PBL.
Q26	When compared to the exclusively conventional learning method, PBL is more time-consuming.
Q27	PBL is effective without having any conventional lectures on the subject.
Q28	With PBL, learners become active processors of information.
Q29	PBL helps identify knowledge weak areas for further improvement.
Q30	PBL enables the learners to establish a concrete action plan to achieve their learning goals.
Q31	PBL enhances the practical and clinical application of the ideas.
Q32	PBL helps develop clinical thinking, logical thinking, and abstract concepts.
Q33	PBL fulfills an effective integration between different subjects of basic medical sciences (horizontal integration).
Q34	PBL fulfills an effective integration between basic medical sciences with clinical sciences (vertical integration).
Q35	PBL is a reliable tool for developing scientific reading and writing skills.
Q36	PBL facilitates the development of interpersonal skills.
Q37	PBL facilitates the development of intrapersonal skills.
Q38	With PBL, knowledge activates prior knowledge around a problem, rather than specific subjects.
Q39	PBL allows learners to activate prior knowledge and learn to elaborate and organize their knowledge.
Q40	PBL enhances the retention of knowledge by practice, feedback, and evaluation.
Q41	PBL increases the extent of more related knowledge.

**Table 2:** Sociodemographic characteristics of the participants in the study (n = 118).

		n	(%)
Gender	Female	77	65.3
	Male	41	34.7
Nationality	Portuguese	68	57.6
	Brazilian	37	31.4
	Other	13	11.0
		**Mean**	**SD**
Age	Years	28.7	7.6

**Table 3:** Questionnaire answers (n = 118). For each question, the correspondent median, interquartile range (IQR), minimum, and maximum values are presented.

ID	Item	Median (IQR)	Range (Min–Max)
Q01	PBL interest	4.0 (0)	1–5
Q02	Interactive clinical learning environment	4.0 (0)	1–5
Q03	Use of learning resources for clinical learning	4.0 (0)	1–5
Q04	Achievement of curriculum outcomes	4.0 (1)	1–5
Q05	Understanding of basic concepts	4.0 (0)	1–5
Q06	Clinical exam preparation	4.0 (1)	1–5
Q07	Subject understanding	4.0 (0)	1–5
Q08	Student knowledge responsibility	5.0 (1)	1–5
Q09	Visual/spatial learning in a clinical context	4.0 (1)	1–5
Q10	Auditory learning in a clinical context	4.0 (1)	1–5
Q11	Kinesthetic learning in a clinical context	3.0 (2)	1–5
Q12	Conversion to active lifelong learner in a clinical context	4.0 (2)	1–5
Q13	Learning motivation in a clinical context	4.0 (1)	1–5
Q14	Clinical interest in the lectured lessons	4.0 (1)	1–5
Q15	Ability to find information using the internet/library	4.0 (1)	1–5
Q16	Ability for public speaking	4.0 (2)	1–5
Q17	Time-management skills	4.0 (1)	1–5
Q18	Decision-making skills	4.0 (1)	1–5
Q19	Clinical problem-solving ability	4.0 (1)	1–5
Q20	Development of linguistic skills and self-confidence in a clinical context	4.0 (1)	1–5
Q21	Clinical reasoning ability, in a clinical context	4.0 (1)	1–5
Q22	Comparison of the PBL hybrid system with the conventional method	4.5 (1)	1–5
Q23	Comparison of knowledge thoroughness gain	3.0 (2)	1–5
Q24	Comparison of relevance and interest	4.0 (2)	1–5
Q25	Comparison of understanding of objectives	4.0 (1)	1–5
Q26	Time consumption comparison	3.0 (2)	1–5
Q27	Effectiveness comparison	3.0 (2)	1–5
Q28	Active processing of information	4.0 (1)	1–5
Q29	Identification of weakness areas	4.0 (1)	1–5
Q30	Establishment of a concrete action plan for the achievement of learning goals	4.0 (1)	1–5
Q31	Practical and clinical application of ideas	4.0 (1)	1–5
Q32	Development of clinical thinking, logical thinking, and abstract concepts	4.0 (0)	1–5
Q33	Horizontal integration effectiveness	4.0 (1)	1–5
Q34	Vertical integration effectiveness	4.0 (0)	1–5
Q35	Development of scientific reading and writing skills	4.0 (1)	1–5
Q36	Development of interpersonal skills	4.0 (1)	1–5
Q37	Development of intrapersonal skills	4.0 (1)	1–5
Q38	Prior knowledge activation around a problem	4.0 (0)	1–5
Q39	Knowledge organization	4.0 (1)	1–5
Q40	Knowledge retention by practice, feedback, and evaluation	4.0 (0)	1–5
Q41	Extension of related knowledge	4.0 (1)	1–5

**Table 4:** Items overall distribution among components, after principal component analysis (PCA), according to the rotated component matrix, via Varimax with the Kaiser normalization method (cumulative variance (%) = 73.644, Kaiser–Meyer–Olkin (KMO) value = 0.902).

Component	Item	Factor Loadings	Eigenvalue	Variance (%)	Communality
Component 1	Learning motivation in a clinical context (Q13)	0.773	19.251	46.955	0.824
	Clinical interest in the lectured lessons (Q14)	0.762			0.781
	Comparison of relevance and interest (Q24)	0.654			0.671
	Clinical problem-solving ability (Q19)	0.625			0.842
	Comparison of understanding of objectives (Q25)	0.623			0.707
	Effectiveness comparison (Q27)	0.601			0.568
	Comparison of knowledge thoroughness gain (Q23)	0.560			0.700
	Establishment of a concrete action plan for the achievement of learning goals (Q30) *	0.498			0.664
	Identification of weakness areas (Q29)	0.463			0.625
Component 2	PBL interest (Q01)	0.700	2.299	5.607	0.727
	Use of learning resources for clinical learning (Q03)	0.697			0.732
	Clinical exam preparation (Q06)	0.693			0.771
	Achievement of curriculum outcomes (Q04)	0.671			0.763
	Understanding of basic concepts (Q05)	0.671			0.691
	Interactive clinical learning environment (Q02)	0.666			0.733
	Subject understanding (Q07)	0.646			0.683
	Extension of related knowledge (Q41)	0.525			0.765
	Knowledge retention by practice, feedback, and evaluation (Q40)	0.486			0.733
	Prior knowledge activation around a problem (Q38) *^1^	0.406			0.664
Component 3	Ability for public speaking (Q16)	0.860	1.981	4.833	0.800
	Development of linguistic skills and self-confidence in a clinical context (Q20)	0.807			0.828
	Ability to find information using the internet/library (Q15)	0.650			0.592
	Time-management skills (Q17)	0.632			0.685
	Decision-making skills (Q18)	0.611			0.696
	Clinical reasoning ability in a clinical context (Q21)	0.516			0.800
	Development of scientific reading and writing skills (Q35)	0.470			0.707
	Establishment of a concrete action plan for the achievement of learning goals (Q30) *^1^	0.454			0.664
Component 4	Horizontal integration effectiveness (Q33)	0.769	1.632	3.980	0.854
	Vertical integration effectiveness (Q34)	0.671			0.729
	Practical and clinical application of ideas (Q31)	0.605			0.769
	Development of clinical thinking, logical thinking, and abstract concepts (Q32)	0.549			0.741
	Knowledge organization (Q39)	0.513			0.835
	Prior knowledge activation around a problem (Q38) *	0.418			0.664
	Comparison between the PBL hybrid system with the conventional method (Q22)	0.418			0.555
Component 5	Visual/spatial learning in a clinical context (Q09)	0.711	1.598	3.899	0.748
	Conversion to active lifelong learner in a clinical context (Q12)	0.754			0.743
	Kinesthetic learning in a clinical context (Q11)	0.753			0.808
	Auditory learning in a clinical context (Q10)	0.504			0.713
Component 6	Development of interpersonal skills (Q36)	0.643	1.316	3.210	0.865
	Development of intrapersonal skills (Q37)	0.587			0.839
Component 7	Student knowledge responsibility (Q08)	0.841	1.104	2.692	0.795
	Active processing of information (Q28)	0.522			0.750
Component 8	Time consumption (Q26)	0.932	1.012	2.468	0.891

* Question assigned to the corresponding component; *^1^ question not assigned to the corresponding component.

## Data Availability

The data used to support the findings of this study are available from the corresponding author (L.P.) upon request.
